# Adherence to low carbohydrate diets and non-alcoholic fatty liver disease: a case control study

**DOI:** 10.1186/s40795-022-00625-5

**Published:** 2022-11-29

**Authors:** Fatemeh Yaghoubi, Mina Darand, Aazam Ahmadi Vasmehjani, Zahra Darabi, Nasir Talenezhad, Farhang Mirzavandi, Mahdieh Hosseinzadeh

**Affiliations:** 1 Department of Clinical Biochemistry, School of Medicine, Shahid Sadoughi University of Medical Sciences, Yazd, Iran; 2 Nutrition and Food Security Research Center, Shahid Sadoughi University of Medical Sciences, Yazd, Iran; 3 Department of Clinical Nutrition, School of Nutrition and Food Science, Isfahan University of Medical Sciences, Isfahan, Iran; 4 Department of Nutrition, School of Public Health, Shahid Sadughi University of Medical Sciences, Yazd, Iran

**Keywords:** Diet, Carbohydrate-Restricted, Fatty Liver, Non-alcoholic fatty liver disease

## Abstract

**Background:**

Non-alcoholic fatty liver disease (NAFLD) is defined as the excessive accumulation of fat in the liver cells of people who do not drink alcohol. The aim of study is investigated the association between low carbohydrate diets (LCDs) and NAFLD.

**Methods:**

This age and gender-matched case–control study was conducted on 120 patients newly diagnosed with NAFLD and 120 adults without NAFLD. Diagnosis of NAFLD based on laboratory tests and abdominal ultrasound. Low carbohydrate diets score calculated on the percentage of energy as carbohydrate, fat, and protein. Participants in the highest rank intake of fat and protein and lowest intake of carbohydrate received 10 points. Multivariable logistic odds ratio was used for examine the relation between LCDs and NAFLD.

**Results:**

This study showed subjects in the highest tertile of LCD has more intake of zinc and vitamin B12 compare to lowest. Also, intake of protein (*p* = 0.02) carbohydrate (*p* < 0.02) and cholesterol (*p* = 0.02) were significantly higher in patient with NAFLD compare to control subjects. There was no significant association between LCD and risk of NAFLD (OR: 1.36; 95% CI: 0.97–1.92; P-trend = 0.13) in crude and adjusted (OR: 1.31; 95% CI: 0.84–2.04; P-trend = 0.23) model.

**Conclusion:**

However, we showed that intake of protein, carbohydrate and cholesterol are higher in NAFLD, but our results of study showed that LCDs with higher proportion intakes of protein and fat was not associated with NAFLD. Further prospective studies are required for confirm these associations.

## Background & objectives

Nonalcoholic fatty liver disease (NAFLD) is defined as the accumulation of fat in more than 5% of hepatocytes of people whose daily alcohol intake is less than 30 gr in men and less than 20 gr in women [1, 2]. NAFLD can progress to a spectrum of conditions including non-alcoholic steatohepatitis (NASH), which can lead to fibrosis as well as cirrhosis and hepatocellular carcinoma (HCC) [3]. Its prevalence is increasing rapidly, affecting approximately 25% of the world's population [4]. The prevalence of NAFLD is estimated to be around 33.9% in Iran [5]. NAFLD imposes a heavy economic burden on the health system and reduces the quality of life if the disease progresses, so finding suitable strategies to prevent and treatment this disease is essential [6].

It seems like diet and nutritional interventions are detrimental to health and cause liver disease. Several cross-sectional studies suggest that Mediterranean diet components have a protective effect on NAFLD [7–9]. A few research have also shown that higher adherence to the Dietary Approaches to Stop Hypertension (DASH) diet is associated with a lower risk of insulin resistance [10], weight loss [11], and NAFLD [12]. On the other hand, a cohort study also demonstrated that people with a higher Western dietary pattern score had a greater risk of developing fatty liver [13].

Low-carbohydrate diets (LCDs) are a type of diet in which the amount of carbohydrates is restricted and the percentage of protein and fat from the total energy intake increases. Its carbohydrate content is less than 26% of the total daily energy intake or less than 130 gm/day [14]. Recently, several studies have examined the effect of LCDs on liver function in patients with NAFLD [15–17]. For example, in a study, an LCD (less than 20 g of carbohydrates) for six months resulted in significant weight loss and improved steatosis, fibrosis, and inflammation [15]. Another study reported that the LCD decreased liver volumes and liver fat content [16]. On the other hand, the results of a meta-analysis study in 2016 showed that although LCD reduces the fat content of the liver, it does not have a significant effect on the level of liver enzymes [17]. Since obesity, hyperlipidemia, and insulin resistance are major risk factors for liver disease, possible mechanisms of a LCD include weight loss, decreased liver fat content, and decreased insulin resistance [18–21]. Due to the increased prevalence of NAFLD in communities and inconclusive evidence of the efficacy LCDs in the treatment of NAFLD, the present study was conducted to investigate the association between NAFLD and LCDs.

## Material and methods

### Study design and participants

For this case–control study, 120 patients with NAFLD based on laboratory tests and abdominal ultrasound were diagnosed from those who referred to the academic liver disease clinics of Shahid Sadoughi University of Yazd Medical Sciences, from October 2017 to March 2019 by non-probability sampling method. Eligibility criteria included men and women aged 20 to 69 years, body mass index [BMI] > 25 kg/m^2^ and not consume alcoholic beverages. Exclusion criteria were use of hepatotoxicity-inducing drugs (tamoxifen, steroids, amiodarone, having cardiovascular diseases, coronary artery disease, congestive heart disease), diabetes type 1, chronic B or C hepatitis virus infections, cancer, Wilson's disease, hemochromatosis, biliary diseases or cirrhosis, and other liver diseases and history of having a special diet. Abdominal ultrasound was performed by a radiologist with the same device for all participants were then divided into two groups. Liver steatosis was detected by assessing the brightness of the echo image. Abdominal ultrasound in the cases could not detect the accumulation of liver fat when it was less than 33% of the total liver weight. Therefore, they were classified as controls [22]. Diagnosis of NAFLD implemented to recommendations the American College of Gastroenterology and the American Gastroenterological Association [23].

One hundred twenty people without NAFLD were selected after matching for age and sex from the same clinic and over the same time period. The study sample size was calculated as 240 using α = 0.05 and test power of 90% [24], considering a significant odds ratio (OR) of 1.45 [25]. In addition to STROBE Statement, all methods were carried out in accordance with relevant guidelines and regulations [26].

### Assessment of dietary intake

Dietary intakes and nutritional supplements were assessed through a validated FFQ (Food Frequency Questionnaire) consisting of 178 food items which was modified version of a previously validated 168-item FFQ [27]. Additional 10 questions relating to consumption of Yazd-specific food items were added to the original FFQ that were collected by trained interviewer. Amount and frequency of usual consumption of each food item daily (once to four times, five to seven times, seven to nine times, 10 times and more), weekly (once, two to four times, five to six times), and monthly (never or less than once, one to three times) in the past year was asked by trained interviewer who blinded to the participants' categorization in the case or control group. A photo book was used as a reference, so that participants could estimate the portion size of foods accurately. Portion sizes were converted to grams using guidelines of household scales [28]. If more than 20% from questionnaire of FFQ was not answered, subject was removed from analysis. 

### Calculation of the low-carbohydrate-diet score

Participants were divided into deciles based on the percentage of energy intake from fat, protein and carbohydrate. For fat and protein, participants in the highest decile protein and fat intake received a score 10, in the ninth decile received a 9 and so on up to the lowest decile which received a score 0. On the other hand, subjects intake the lowest carbohydrate, received a score 10 and those who consumed the highest received a score 0. Finally, score ranged from 0 (the highest carbohydrate intake and the lowest fat and protein intakes) to 30 (the highest protein and fat intakes and the lowest carbohydrate intake) [29]. Therefore, participants with higher score adherence to a low-carbohydrate diet [30].

### Anthropometric and physical activity measurements

Weight was determined in light clothes with minimum clothing and in standing position on scale using Omron Digital Scale, Model BF511 with an accuracy of 0.1 kg. Height was measured using a tape meter with 0.1 cm precision in standing position without shoes. BMI was calculated by dividing weight in kilograms to height in meters squared. Waist circumference (WC) measurement was performed using non-stretch tape meter, while it is middle of the iliac crown and lowest rib in the standing position with accuracy 0.5 cm and so was hip circumference measurement from the largest part of the buttocks. The International Physical Activity Questionnaire was used to assess physical activity [31].

### Assessment of other variables

On the day of visit, blood pressure was measured by a digital pressure indicator (Citizen Japan Company, CH456 model) after 10 min of rest on the chair and without any intense physical activity. Data related to age, gender, education (High school, Diploma, Associate Degree, Bachelor's and higher), job (Housewife, Employee, Free job), history of diabetes (No, Yes), tobacco and alcohol (No, Yes: used as an exclusion criterion), as well as medications and dietary supplements (No, Yes) were collected.

### Statistical analysis

Using SPSS software (SPSS, Chicago, IL, USA) version 20 was performed all statistical analyses. Independent t-test and Chi-square test conducted to compare continuous and non-continuous variables between two groups, respectively. Characteristics of participants compared throughout deciles of low carbohydrate diet score using analysis of variance for continuous variables and Chi-square test for categorical variables. A multiple logistic regression model was exerted by odds ratio and 95% confidence interval (OR (95%CIs)) to examine the relationship between fatty liver and low carbohydrate diet score in each tertile and it overall trend in crude and multivariable-adjusted models.

## Results

### Characteristics of the study population

The characteristics of the study participants in case or control groups are presented in Table 1. The mean age of the participant was 43.89 years. Gender, Job, Participant’ educational level, diabetes and physical activity was not significantly differed between case and control group. There were significant differences between BMI in case and control group. Patient with NAFLD were in higher BMI compared to control group.

**Table 1:** characteristics of the study participants across base on case and control groups

Variable	Control group	Case group	*p*_value*
**Age (mean ± SD)**	43.52(12.14)	44.22(10.35)	0.64
**BMI (mean ± SD)**	25.26(3.94)	30.39(4.10)	< 0. 01
**Gender N (Percent)**			0.92
Male	45(44.1)	50(43.5)	
female	57(55.9)	65(56.5)	
**Job N(Percent)**			0.20
Housekeeper	40(39.2)	59(51.3)	
Employee	33(32.4)	30(26.1)	
Freelance	29(28.4)	26(22.6)	
**Education N (Percent)**			0.31
High school	34(33.3)	44(38.3)	
Diploma	22(21.6)	33(28.7)	
Bachelor	5(4.9)	5(4.3)	
Masters degree and higher	41(40.2)	33(28.7)	
**Diabetes N (Percent)**	26(25.5)	67(58.3)	0.47
**Physical activity N(Percent)** Yes			0.11
	70(68.6)	67(58.3)	
No	32(31.4)	48(41.7)	

*BMI* Body mass index

^*^independent t test used for quantitative variable and chi-square test for qualitative variable

### Dietary intakes of participants

Intake of macronutrient and some micronutrient of all participant across tertiles of LCD presented in Table 2. Intake of zinc, B12 and vitamin C was significantly differed across tertiles of LCD. Subjects in third tertile has consumed higher zinc and vitamin B12 and lower vitamin C compare to subjects in first tertile (*p* < 0. 01). Intake of some food group and macronutrient in case and control group were presented in Table 3. Intake of carbohydrate (*p* < 0.02), protein (*p* = 0.02) and Cholesterol (*p* = 0.02) were significantly higher in patient with NAFLD compare to health subjects.

**Table 2:** Intake of macronutrient and some micronutrient participant across tertiles of LCD

Variable*	T1	T2	T3	p**
**Energy intake(kcal)**	2184.27 ± 671.49	2198.73 ± 629.29	2122.61 ± 805.11	0.78
**Carbohydrate(gr)**	353.99 ± 110.31	317.08 ± 97.90	263.06 ± 106.37	< 0.001
**Whole grain(gr)**	51.75 ± 40.42	45.97 ± 35.18	39.37 ± 33.30	0.13
**Refined grain(gr)**	262.29 ± 51.75	243.80 ± 45.97	191.52 ± 125.98	< 0.01
**Protein(gr)**	78.60 ± 26.65	92.36 ± 35.18	103.49 ± 45.65	< 0. 001
**Fat(gr)**	61.12 ± 20.01	75.37 ± 23.92	84.39 ± 33.49	< 0. 001
**Calcium(mg)**	789.87 ± 289.04	755.05 ± 258.76	775.81 ± 334.15	0. 82
**Zinc(mg)**	8.95 ± 3.07	10.03 ± 3.58	11.14 ± 4.45	< 0. 01
**Iron(mg)**	47.47 ± 82.26	60.70 ± 100.70	38.70 ± 53.79	0. 25
**VitaminB12(µg)**	3.49 ± 1.63	4.16 ± 1.89	5.20 ± 0.09	< 0. 01
**Folate(µg)**	320.48 ± 131.19	286.03 ± 108.94	279.31 ± 116.19	0. 08
**ViaminB6(mg)**	2.02 ± 0.73	2.03 ± 0.83	2.06 ± 1.02	0. 95
**Vitamin C(mg)**	234.10 ± 119.47	184.82 ± 92.06	169.02 ± 198.20	< 0. 01

*LCD* Low carbohydrate diet

^*^independent t test used for quantitative variable and chi-square test for qualitative variable

^*^Value are presented as mean ± SD

^**^one-way analysis of variance for analysis

**Table 3:** Intake of some food group and macronutrient in case and control group

variable	Group	Mean	Std. Deviation	P*
Energy(kcal)	control	2050.1207	722.69976	0.01
	case	2274.0845	670.25926	
Carbohydrate(gr)	control	288.4438	106.41338	< 0.01
	case	331.1336	111.12786	
Fat(gr)	control	71.1171	28.90024	0.18
	case	76.1290	27.05606	
Protein(gr)	control	85.3812	36.11251	0.02
	case	97.1513	38.74577	
Cholesterol(mg)	control	300.1015	138.79802	0.02
	case	322.1143	165.23739	
SAFA(gr)	control	21.7453	9.06459	0.65
	case	22.2709	8.11072	
MUNO(gr)	control	21.4873	9.14886	0.23
	case	22.9847	9.43529	
EPA(gr)	control	0.0233	0.06911	0.91
	case	0.0225	0.02442	
DHA(gr)	control	0.0607	0.17992	0.92
	case	0.0590	0.06377	
Refined grains(gr)	control	222.2804	140.92292	0.32
	case	241.4181	141.95683	
Whole grains(gr)	control	46.1138	38.03616	0.86
	case	45.2361	35.34356	
legumes(gr)	control	30.4115	19.92618	0.29
	case	33.6072	24.51574	

*SFA* Saturated fatty acid, *PUFA* Poly unsaturated fatty acid, *MUFA* Mono unsaturated fatty acid

^*^independent t test used

### Low carb diet and NFLD

Crude and multivariate-adjusted odds ratios for NAFLD across tertiles of LCD are listed in Table 4. There was no significant association between high LCD and risk of NAFLD (OR: 0.79; 95% CI: 0.41–1.54; P-trend = 0.49) in crude model. No significant association was remined after adjusting potential confounders including energy intake, physical activity, job, level of education, BMI, diabetes, sex and age (OR: 0.81; 95% CI: 0.35–1.89; P-trend = 0.65).

**Table 4:** Odds ratio NAFLD across tertiles of LCD (Multivariable-adjusted odds ratios and 95% confidence intervals)

NAFLD	T1	T2	T3	*P_value	P trend
Crude	1.00	1.49(0.77_2.89)	0.79 (0.41_1.54)	0.50	0.49
Adjusted model1	1.00	1.76 (0.87_3.56)	0.88 (0.44_1.78)	0.73	0.75
Adjusted model2	1.00	1.53(0.66_3.51)	0.81 (0.35_1. 89)	0.63	0.65

NAFLD: Nonalcoholic fatty liver disease, LCD: Low carbohydrate diet

1. Adjusted for energy intake, educational level, physical activity, sex, age, job and diabetes

2. Adjusted for model 1 and BMI

^*^Third tertile compare to first tertile

## Discussion

This is an age and gender-matched case control study in which the relationship between LCD and the risk of NAFLD was assessed. It is expected that the mentioned diet can decrease the odds of NAFLD. Our data have not shown any significant relationship between LCD and NAFLD. However, patient with NAFLD consumed higher intake of protein, carbohydrate and cholesterol compare to control subjects.

There is some controversy result over the effect of LCD and NAFLD in published articles. This is maybe due to a lack of consensus on the definition of the low-carbohydrate diet; however, based on the amount of carbohydrate intake, three categories can be classified; [1] the “ketogenic diet” or a “very-low-carbohydrate diet” could be defined if the carbohydrate intake rate (as a ratio of carbohydrate intake to the total calorie intake per day) is < 10%, or < 30 g/day, [2] “low-carbohydrate diet” contains the carbohydrate intake rate between < 26%, or > 30 g/day and [3] and when the carbohydrate intake ratio is between 26 and 45%, or > 130 g/day, it is classified as “reduced carbohydrate diet” or the “moderate carbohydrate diet” [32]. While, another study categorized low, moderate, and high-carbohydrate diets with 20 g/d or less, 60 g/d or less, and more than 60 g/d, respectively [33].

In general, in Middle Eastern countries, including Iran, the average consumption of carbohydrates is high, due to a food pattern with high consumption of cereal, compared to non-Middle Eastern countries. Although, in the current study adherence to LCD was assessed, the intake of participants was important. Across total consumption of participants, mean intake of carbohydrate, fat and protein was calculated in each tertile or quartile, However, the amount of carbohydrate intake in the current study was < 30% or < 130 g/day. LCD has been defined in the interventional studies, while in the observational studies score of LCD was calculated across previous studies. For example, in the previous observational studies that investigated the association between low carbohydrate diet score and other diseases, intake of carbohydrate diet is higher than 130 g in highest N tertile [34, 35].

The finding of Benjaminov’s study have shown reduced liver fat content and liver size after 4 weeks treatment with a very low carbohydrate diet (30 g/day) [36]. While, the result of a recently published meta-analysis which has assessed the impact of LCD in NAFLD patients, did not show any significant effect of LCD on the improvement of transaminases and hepatic fat content in NAFLD [37]. Therefore, many researches have more emphasis on low calorie diet rather than LCD in similar calorie intake [33, 37, 38].

Scribner et al. have studied the glycemic index of the carbohydrates in their research. They showed that both of rapidly absorbed or slowly absorbed of carbohydrates caused NAFLD in mice with similar body weight and controlled for dietary factors. Although Plasma insulin, body adiposity and Triacylglyerol were higher in the rapidly absorbed carbohydrates group [39]. Since, there is a strong association between body weight and metabolic syndrome with NAFLD as well as lack of clinical trials about the impact of a LCD on NAFLD, therefore, the effects of a LCD on body weight and metabolic syndrome will be discussed. A recent published systematic review containing 107 studies observed a principal association between weight loss in participants who use LCD with increased diet duration and decreased caloric intake but not with reduced carbohydrate content [33]. While, Fraser et al. in a quasirandomized controlled study found a significantly decreased in the levels of alanine aminotransferase in the low-carbohydrate/low-glycemic-index diet (35%) group compared to high-carbohydrate/low-glycemic-index diets [40]. It seems unreasonable if long-term use of LCD (∼ < 40% calories) has been used, therefore researches on moderate carbohydrate diet (∼40%–50% calories) have been performed [38]. Golay et al. followed two group of patients for 12 weeks who received a hypocaloric diet with a 25% carbohydrate or a 45% carbohydrate. At last, they did not observe significantly differences between the loss of adipose tissue and the mean weight loss [41]. A few studies have shown developing of benign fatty liver to Non-alcoholic steatohepatitis (NASH) following consumption of a conventional high-carbohydrate diet. Also, such studies have demonstrated an association between high carbohydrate diet with increased insulin resistance and obesity [42]. A retrospective study conducted by Solga et al. through a standardized 24-h food recall on obese patients who presented for bariatric surgery. After evaluating food intake for macronutrients and total calories, they observed that higher carbohydrate intake was meaningfully depended with higher rate of inflammation compared to higher fat intake [43].

An important factor which can trigger the beginning of NAFLD is excessive hepatic lipogenesis and hypertriglyceridemia which in turn is as a result of high intake of carbohydrates such as glucose and fructose [44–50]. Therefore, a possible strategy for reducing NAFLD is a reduction in sugar consumption [15]. Other factors including dietary fat and proteins and choline and methionine content also individual’s health status would be effect on the effectiveness of LCD and NAFLD. Indeed ketogenic diet encompassing very high in fat, very low in carbohydrate and moderate in protein content increases intrahepatic triglyceride (IHTG) concentrations through induction of hepatic insulin resistance [51–54]. Because some conditions such as insulin resistance plays principal role in increasing hepatocyte carbohydrate through diversion of glucose to the liver [55]. Choline is an essential constituent for synthesis of neurotransmitter acetylcholine as well as mitochondrial membranes that ultimately is metabolized to phospholipids or S adenosyl-L-methionine (SAM), a methyl donor [56] which its deficiency heightens the risk of NAFLD by reduced secretion of VLDL and increased fatty acids uptake [57]. Diverse individual’s health gives different responses to a same diet. As Hellerstein and his colleagues reported that high fat intake diminishes hepatic de nove lipogenesis (a major pathway in increasing hepatic triglyceride content in NAFLD patients) while high carbohydrates intake increases hepatic de novo lipogenesis in obese individuals with hyperinsulinemia compared to obese individuals with normal insulin [58]. Furthermore, individual’s heritage may be contributing in the progression of NAFLD [59–61]. Hepatic fibroblast growth factor 21 (FGF21), a major factor in regulating hepatic metabolism, glucose, fatty acid and insulin resistance, are increased in patients with NAFLD [62–65]. Such results strongly suggest that much cautions and precise dietary recommendations should be considered basis on the individual’s health.

Selection the sample from the same clinic as well as matching them in terms of age and sex and collection of FFQ by trained interviewer who blinded to the participants' categorization in the case or control group, which minimized the reporting error, were among the strengths of this study. Also, we used new diagnosed individuals with NAFLD (Incident case) as case group. Various confounders associated with fatty liver and also dietary pattern were adjusted, particularly the total energy intake. On the other hand, some factors lead to show causal link between LCD and the risk of NAFLD such as the type of the study design, the effect of unknown confounding factors in the statistical methods, use of a moderate carbohydrates diet or the use of tertiles that could lead to bias and inefficiency. It is suggested that some surveys such as cohort and controlled trials would be assessed the effect of LCD on NAFLD.

## Conclusion

NAFLD is the most prevalent chronic liver disease in the world which is identified by excessive accumulation of fat in the liver cells of people who do not drink alcohol. Pharmacologic interventions are limited owing to their adverse effects and low efficacy. Therefore, lifestyle intervention and weight loss remains as a good choice for treatment of NAFLD. Exercise and dietary modification such as hypocaloric diets either in carbohydrate content or macronutrient composition lead to weight loss. Also, studies have demonstrated the effectiveness of low- and very-low-carbohydrate diets without total calorie restriction in short-term weight loss. While, other studies have shown improvement in insulin resistance, liver histology, and other factors link to metabolic syndrome, that are strongly associated with NAFLD.

As a result, in this study we assessed the association between LCDs and NAFLD. Although our result has shown that the intake of protein, carbohydrate and cholesterol are higher in NAFLD patients, but LCD diet with higher proportion intakes of protein and fat was not associated with NAFLD. Consequently, Further prospective studies are needed for confirm these associations.

## Data Availability

The datasets used and/or analysed during the current study available from the corresponding author on reasonable request. Because in the protocol from which this data was extracted, it is approved that the data will be provided if an agreement is signed between the parties.
